# The potential risk of enzootic *Trypanosoma cruzi* transmission inside four training and re-training military battalions (BITER) in Colombia

**DOI:** 10.1186/s13071-021-05018-4

**Published:** 2021-10-09

**Authors:** Omar Cantillo-Barraza, Jeffer Torres, Carolina Hernández, Yanira Romero, Sara Zuluaga, Camilo A. Correa-Cárdenas, Giovanny Herrera, Omaira Rodríguez, María Teresa Alvarado, Juan David Ramírez, Claudia Méndez

**Affiliations:** 1 Grupo de Investigación en Enfermedades Tropicales del Ejército (GINETEJ), Laboratorio de Referencia E Investigación, Dirección de Sanidad Ejército, Bogotaá, Colombia; 2 Centro de Investigaciones en Microbiología y Biotecnología—UR (CIMBIUR), Facultad de Ciencias Naturales, Universidad del Rosario, Bogotá, Colombia; 3 Centro de Tecnología en Salud (CETESA), Innovaseq SAS, Bogotá, Colombia; 4 Grupo Biología Y Control de Enfermedades Infecciosas (BCEI), Universidad de Antioquia, Calle 70 No. 52-21, Medellín, Colombia

**Keywords:** Chagas disease, *Trypanosoma cruzi*, Colombia, Colombian National Army, Non-domiciliated triatomines, *Rhodnius pallescens*, *Rhodnius prolixus*, Entomological and mammal surveillance, Reservoirs, *Didelphis marsupialis*

## Abstract

**Background:**

Colombia’s National Army is one of the largest military institutions in the country based on the number of serving members and its presence throughout the country. There have been reports of cases of acute or chronic cases of Chagas disease among active military personnel. These may be the result of military-associated activities performed in jungles and other endemic areas or the consequence of exposure to *Trypanosoma cruzi* inside military establishments/facilities located in endemic areas. The aim of the present study was to describe the circulation of *T. cruzi* inside facilities housing four training and re-training battalions [Battalions of Instruction, Training en Re-training (BITERs)] located in municipalities with historical reports of triatomine bugs and Chagas disease cases. An entomological and faunal survey of domestic and sylvatic environments was conducted inside each of these military facilities.

**Methods:**

Infection in working and stray dogs present in each BITER location was determined using serological and molecular tools, and *T. cruzi* in mammal and triatomine bug samples was determined by PCR assay. The PCR products of the vertebrate *12S rRNA* gene were also obtained and subjected to Sanger sequencing to identify blood-feeding sources. Finally, we performed a geospatial analysis to evaluate the coexistence of infected triatomines and mammals with the military personal inside of each BITER installation.

**Results:**

In total, 86 specimens were collected: 82 *Rhodnius pallescens*, two *Rhodnius prolixus*, one *Triatoma dimidiata* and one *Triatoma maculata.* The overall *T. cruzi* infection rate for *R. pallescens* and *R. prolixus* was 56.1 and 100% respectively, while *T. dimidiata* and *T. maculata* were not infected. Eight feeding sources were found for the infected triatomines, with opossum and humans being the most frequent sources of feeding (85.7%). Infection was most common in the common opossum *Didelphis marsupialis*, with infection levels of 77.7%. Sylvatic TcI was the most frequent genotype, found in 80% of triatomines and 75% of *D. marsupialis.* Of the samples collected from dogs (*n* = 52), five (9.6%; 95% confidence interval: 3.20–21.03) were seropositive based on two independent tests. Four of these dogs were creole and one was a working dog. The spatial analysis revealed a sympatry between infected vectors and mammals with the military population.

**Conclusions:**

We have shown a potential risk of spillover of sylvatic *T. cruzi* transmission to humans by oral and vectorial transmission in two BITER installations in Colombia. The results indicate that installations where 100,000 active military personnel carry out training activities should be prioritized for epidemiological surveillance of Chagas disease.

**Graphical abstract:**

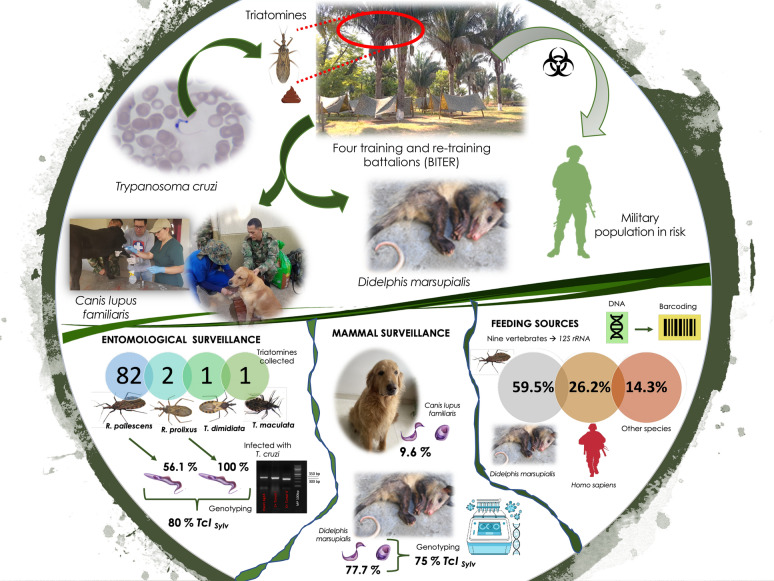

**Supplementary Information:**

The online version contains supplementary material available at 10.1186/s13071-021-05018-4.

## Background

Chagas disease (CD) caused by the parasite *Trypanosoma cruzi,* affects about six million people in Latin America [1], and it has been estimated that 1.67–2% of the population of Colombia are infected with this parasite [2]. *Trypanosoma cruzi* is transmitted to humans mainly by triatomine vectors (Hemiptera: Reduviidae) through contact of the skin and mucous membranes with feces and other secretions of infected insects [3]. However, this parasite may be also transmitted by blood transfusion, congenitally, organ transplantation, laboratory accidents and ingestion of food/drinks contaminated with *T. cruzi* [4].

*Trypanosoma cruzi* is a hemotropic protozoan that infects around 158 mammalian species. It exhibits broad intraspecific genetic diversity and is categorized into at least six discrete typing units (DTUs) [5, 6], one of which, TcBat, is an emerging genotype. *Trypanosoma cruzi* DTU I (TcI) is the most prevalent DTU in the region north of the Amazon basin, and it can be further subdivided into two major genotypes, namely domestic (TcI_Dom_) and sylvatic (TcI_Sylvatic_) [7, 8]. The former is generally associated with the domestic transmission cycle in specific areas, and the latter is frequently involved in enzootic transmission and oral outbreaks [9, 10].

Domestic transmission by domiciliated species of the *Triatoma* and *Rhodnius* genera has traditionally been responsible for higher transmission rates of CD in areas of high economic and social deprivation in Central and South America [11–14]. However, following the systematic control of CD intradomestic vectors in Brazil, Argentina, Paraguay, Chile, Guatemala, Nicaragua and Colombia, newer scenarios mediated by non-domiciled triatomines have come to the fore in terms of *T. cruzi* transmission dynamics [15–25]. In these scenarios, new cases occurr more commonly in residents of relatively older age, and *T. cruzi* transmission is associated with environmental and ecological characteristics mediated by reinfestation of peridomestic native species of triatomines. In addition, there have been outbreaks due to oral transmission in places where sylvatic triatomines invade houses and where there is a positive selection of generalist species with high competence as reservoirs [17, 26–29]. The development of fishing, hunting and agricultural activities that bring people closer to the enzootic cycle [16, 30, 31] and working, educational and social activities where people share the same food increase the risk of spillover of enzootic transmission to humans [32].

Colombia’s National Army has approximately 400,000 soldiers and is the largest military institution in terms of manpower in Colombia. The soldiers actively protect Colombia’s sovereignty in border areas and patrol and maintain public order in all municipalities, including those with enzootic transmission of *T. cruzi*. Acute cases of CD related to vectorial and oral transmission as well as chronic cases among active military personnel in military installations are usually reported in the National Public Health Surveillance System (SIVIGILA) [33–35]. A recent study of *T. cruzi* infection among the active military population found that around 70.5% of the evaluated population were born in municipalities that have reported infected triatomines [35–37]. In this context, it is possible that military personnel exposed to different scenarios of CD transmission could converge in battalions or cantonment centers, combined with the existence of triatomines bugs and reservoirs. This scenario could result in a potential risk of transmission inside military establishments, but to date there has been no study on this potential scenario.

Battalions of Instruction, Training en Re-training (BITERs: acronym in Spanish) are an essential military establishment within the organizational structure of Colombia’s National Army. In these units, many professional soldiers congregate for training activities, which also include the initial training for recently drafted recruits as part of the mandatory military service in Colombia. Due to the epidemiological relevance of BITERs in terms of *T. cruzi* infection spillover, the aim of this study was to evaluate the potential of *T. cruzi* transmission inside four BITER units located in endemic areas of Colombia.

## Methods

### Study area

**Fig. 1:**
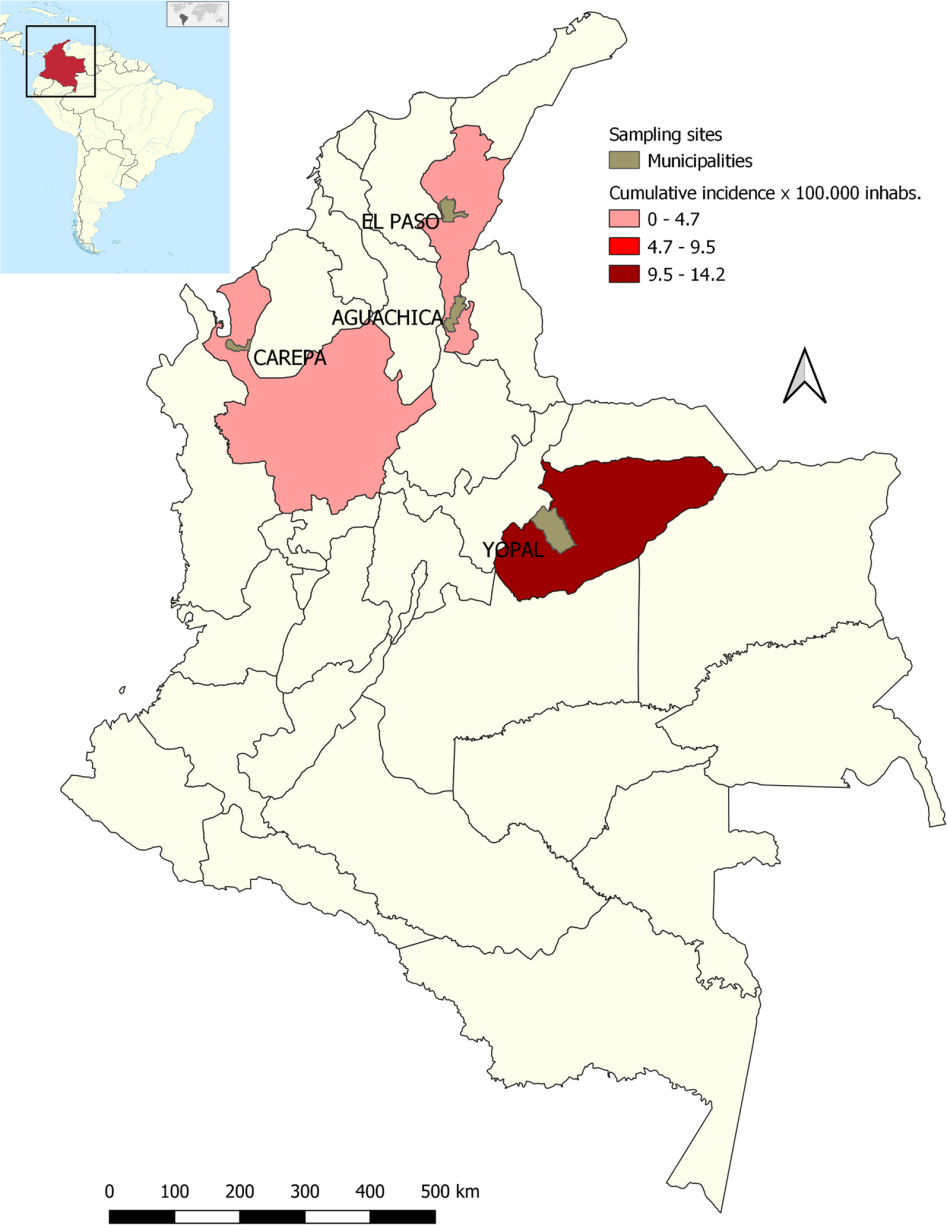
Map showing the locations (3 departments) of the four BITERs of the Colombia National Army that were sampled between 2019 and 2020

This study was carried out between October 2019 and March 2020 in four BITERs located in four endemics municipalities: BITER 16, Yopal (5°29′31″N, 73°29′12″W) in the department of Casanare; BITER 17, Carepa (5°19′50″N, 72°23′26″W) in the department of Antioquia; BITER 5, Aguachica (8°11′46″N, 73°36′47.32″W) and BITER 10, La Loma (9°35′43.98″N, 73°26′42.38″W) both in the department of Cesar (Fig. 1). These BITERs receive different units of the National Army that are gathered together in each department for training and re-training activities and consist of around 2,500 Army members that inhabit each military installation, carrying out camping activities and receiving food rations prepared inside the battalion.

### Insect collection

The entomological study was designed to gather samples in different tropical seasons (rainy and dry). Sample collection during the rainy season was conducted between October and November 2019, and during the dry season samples were collected from February to March 2020. Searches for triatomine insects were carried inside bedrooms and lodgings with permission and cooperation of the soldiers and military and governmental officials. We also examined all palms of *Attalea butyracea* species located < 10 m distance from bedrooms, lodgings and kitchens of each BITER for triatomines following the methodology described by Romaña [38]. Dry and green leaves, organic debris, interfoliaceous meshes and bracts were examined for the presence of triatomines with the help of a ladder. Collected bugs were transported to the BCEI laboratory (University of Antioquia), registered and identified using taxonomic keys [39].

### Molecular detection of *T. cruzi*

Total DNA was extracted from 200 µl of the reservoir host’s blood, hemoculture of mammals and feces of collected triatomines, using a genomic DNA purification kit (Invisorb® Spin Universal Kit; STRATEC Molecular GmbH, Berlin, Germany). *Trypanosoma cruzi* infection was detected by conventional PCR (cPCR) in a total volume of 25 µl containing 40–50 ng of genomic DNA previously quantified using a BioDrop uLite Spectrophotometer (Biochrom US Inc., Holliston, MA, USA), 1× of buffer, 0.04 mM of dNTP, 1.5 mM MgCl_2_, 0.4 µM of TCZ1 (5′-GCTCTTGCCCACA(AC)GGGTGC-3′) and TCZ2 (5′-CCAAGCAGCGGATAGTTCAGG-3′) primers [40–43] and 0.05 U of Taq polymerase (Invitrogen, Fisher Thermo Scientific, Waltham, MA, USA). The thermal cycling conditions for amplification of a 188-bp fragment of satellite DNA (SatDNA) were: pre-heating at 95 °C for 15 min; followed by 40 cycles at 95 °C for 10 s, 55 °C for 15 s and 72 °C for 10 s; and then a final extension of 72 °C for 10 min [41]. The amplification products were electrophoresed in a 1.5% agarose gel, stained with SYBR Safe DNA Gel Stain and visualized under UV light using the molecular imager® Gel DOC™ XR+ with Image Lab™ software (Bio-Rad Laboratories Inc., Hercules, CA, USA).

### Genotyping

In positive samples with *T. cruzi* infection, PCR was performed using three different molecular markers to detect the six DTUs and the two types of TcI (TcI_Dom_ and TcI_Sylvatic_). The molecular markers amplified by PCR were two regions of the intergenic miniexon-gene (SL-IR) and ribosomal DNA (rDNA) 24Sα [42, 44, 45]. Green Master Mix GoTaq® (Promega, Madison, WI, USA) was used for amplification of these three targets. All reaction volumes contained 12.5 µl of Master Mix GoTaq® Green, 10 µM of each primer and 5 µl of DNA to which water was added to a final reaction volume of 25 µl. Primers and PCR conditions can given in Additional file 1: Appendix S1. Amplification products were electrophesed in a 1.5% agarose gel, stained with SYBR Safe DNA Gel Stain and visualized under UV light using the molecular imager® Gel DOC™ XR+ and Image Lab™ software (Bio-Rad Laboratories Inc.) based on the sizes of amplicons in each PCR according to the algorithm (Additional file 2: Figure S1) [42].

Samples of the DTU TcI were classified into TcI_Dom_ and TcI_Sylvatic_ genotypes. The reaction mixture consisted of 1× Taq polymerase amplification buffer (100 mM Tris–HCl, pH 8.3; Invitrogen, Thermo Fisher Scientific), 100 mM deoxynucleoside triphosphate solution, 25 mM MgCl_2_ solution, 5 U/µl of Taq polymerase platinum (Invitrogen, Thermo Fisher Scientific), 50 pM of *T. cruzi* mini-exon primers 1Am (5′-TGTGTGTGTATGTATGTG-3′) and 1B (5′-CGGAGCGGTGTGTGCAG-3′) [46]. The thermal cycling profile consisted of an initial denaturation of 94 °C for 4 min; followed by 35 cycles of 94° for 30 s, 55 °C for 20 s and 72 °C for 20 s; and then a final extension of 72 °C for 10 min [9, 47]. Seven strains were used as positive controls: MHOM/CO/01/DA (TcI_Dom_), MHOM/CO/10/GC (TcI_Sylvatic_), TcII (Y), TcIII (CM17), TcIV (YLY), TcV (V) and TcVI (CLBrener).

### Blood-meal sources

Blood-meal sources in all *T. cruzi*-positive triatomines were identified by PCR products of the vertebrate *12S rRNA* gene, which were obtained through amplification of a 215-bp fragment using primers L1085 (5′-CCCAAACTGGGATTAGATACCC-3′) and H1259 (5′-GTTTGCTGAAGATGGCGGTA-3′) [48]. All reaction volumes contained 12.5 µl of Master Mix GoTaq® Green, 10 µM of each primer and 5 µl of DNA to which water was added to a final reaction volume of 25 µl. The thermal cycling profile consisted of an initial denaturation of 94 °C for 4 min; followed by 35 cycles of 94° for 30 s, 57 °C for 15 s and 72 °C for 30 s; and a final extension of 72 °C for 10 min. Amplification products were electrophoresed in a 1.5% agarose gel, stained with SYBR SafeDNA Gel Stain and visualized under UV light using the molecular imager® Gel DOC™ XR + with Image Lab™ software (Bio-Rad Laboratories Inc.). The PCR products were cleaned using ExoSAP-IT® Express PCR Product Cleanup 75001/75002 (Affimetrix, Thermo Fisher Scientific) and then submitted to Macrogen Korea (Seoul, South Korea) for Sanger sequencing (terminal dideoxy method) using the BigDye™ Terminator v3.1 Cycle Sequencing Kit with an AB3730xI automatic sequencer. The resulting sequences in both directions were edited in MEGA X software [49], assembled and checked by eye for all base changes according to the quality of peaks (height, not overlapping and evenly spaced) prior to be submitted to the bsic local alignment search tool for nucleotide databases (BLASTn) of the National Center for Biotechnology Information (NCBI; https://blast.ncbi.nlm.nih.gov) for similarity search, defining each species with percentage identity > 92% and e-value close to 0.00. All new genetic sequences of *12S rRNA* derived from the blood-meal source analysis were deposited and are available in GenBank (https://www.ncbi.nlm.nih.gov/genbank/) under accession numbers OK040172 - OK040196 and OK058390 - OK058403.

### Survey of wild hosts and detection of *T. cruzi* in small mammals

Small mammals were captured using traps (Tomahawk® and Sherman® traps) baited with a mixture of peanuts, bananas, oats and fish. At each BITER, traps were set for 5 nights near sampled palm trees and placed in locations where there had been previous sightings of mammals. The traps were distributed along linear transects, with capture points established 20 m apart from each other. Additionally, mammals present in the palms during wild triatomine collection were captured and anesthetized (50 mg/kg body weight of ketamine, administered by intramuscular injection), and blood samples were collected and stored for DNA extraction and molecular diagnosis. Also, two tubes containing Novy-MacNeal-Nicolle medium medium covered with an liver infusion tryptose (LIT) overlay were inoculated with 0.3–0.6 m of blood. These tubes were examined once weekly for 3 months for the presence of *Trypanosoma* spp.

### Blood sampling and serological diagnostic tests in dogs

Fifty-two dogs (*Canis lupus familiaris*), including 33 military working dogs and 19 creoles, were studied in the four BITERs. For each animal, two 5-ml blood samples were collected from the radial vein into serum and EDTA.K3 vacutainers and stored at 4 °C until processed. For serum extraction, samples were centrifuged at 5000 *g* for 10 min for separation, and the serum was stored at − 20 °C until serological assays were performed. Genomic DNA was extracted from 200 µl of blood from the EDTA.K3 vacutainer using the Genomic DNA Purification Kit (DNeasy Blood & Tissue Kit; Qiagen, Hilden, Germany) according to the manufacturer’s instructions. Total DNA was diluted with 100 µl elution buffer and stored at − 20 °C until molecular diagnosis, as mentioned above.

Anti-*T. cruzi* antibodies (immunoglobulin G [IgG]) in dogs were detected using an enzyme-linked immunosorbent assay (ELISA) and an indirect immunofluorescence antibody test (IFAT). For both techniques, the antigen was prepared from harvested epimastigotes of *T. cruzi* Colombian strains (I.RHO/CO/00/CAS-15.CAS; I. TRI/CO/03/MG-8.MAG), previously characterized as TcI [42]. For the ELISA, a whole lysate extracted from epimastigotes was used as antigen, while for IFAT, antigen was obtained from complete epimastigotes fixed in 1% formaldehyde as reported elsewhere [50]. The detection cutoff was determined as optical absorbance ≥ 0.200 (mean ± standard deviation [SD] of negative control) for the ELISA and sera dilution of ≥ 1/40 for the IFAT, as reported elsewhere [28]. Animals were defined as positive for anti-*T. cruzi* antibodies when the samples were reactive to both tests; the ELISA and IFAT used by Bio-Manguinhos/Fiocruz (Rio de Janeiro, RJ, Brazil) have a 100% sensitivity and 98.7% specificity [28]. Cross-reactions and/or mixed infections of *T. cruzi* and *Leishmania* spp. in serum were also assayed using antigens derived from a mixture of *Leishmania infantum* and *Leishmania panamensis* using IFAT. The IFAT cutoff value adopted for *T. cruzi* infection was 1/40 when IFAT results for *Leishmania* spp were negative; in *Leishmania* spp.-positive dogs, positive *T. cruzi* infection was considered only when the IFAT titer was ≥ 1/80 [50].

### Geospatial analysis

The map of BITER bases was acquired from the National Administrative Department of Statistics (MG-DANE) (https://geoportal.dane.gov.co/servicios/descarga-y-metadatos/descarga-mgn-marco-geoestadistico-Nacional/). All biological data coordinates were captured using a hand-held Global Positioning System (GPS) and recorded in the World Geodetic System 1984 (WGS 84 Datum) geodetic coordinate system. We analyzed the spatial distribution of *T. cruzi*-infected triatomines and mammals inside military installations, and hotspots of potential transmission were geolocated and generated with the QGIS 3.10.9 program [51].

### Data analysis

The statistical association between the number of *Rhodnius pallescens* collected, seasonality (rainy or dry season) and infection rate comparisons were performed using the chi-square test with a *P* < 0.05 considered to be significant. Data analyses were performed using SPSS version 18.0 statistical software (SPSS, IBM Corp., Armonk, NY, USA).

## Results

### Entomological survey, *T. cruzi* infection rate and blood-meal sources

A total of 86 triatomine bugs were collected in three of four BITER locations evaluated during this study, all in palms and wooded areas (Table 1). The species captured were mostly *R. pallescens* (95.3%, *n* = 82), followed by *Rhodnius prolixus* (2.3%, *n* = 2), *Triatoma dimidiata* (1.2%, *n* = 1) and *Triatoma maculata* (1.2%, *n* = 1). No triatomines were found inside bedrooms or lodgings. However, one *R. pallescens* and one *T. dimidiata* were collected by military personnel in BITER 17 (Carepa) in the wooded area. All triatomines collected in palms were located between 3 and 10 meters from the kitchen and bedrooms in BITER 5 (Aguachica) and BITER 16 (Yopal).

**Table 1:** Number of triatomines and mammals in four BITERs of Colombia between 2019 and 2020

BITER	Triatomine species									*Didelphis marsupialis / Proechimys *sp.^ b^				
	*Rhodnius pallescens*	*Rhodnius prolixus*	*Triatoma dimidiata*	*Triatoma maculata*	*Tryanosoma cruzi/ *infection	Tcl_Sylvatic_^a^	TcI_Dom_^a^	Mix	Not identified	*Tryanosoma cruzi* Infection	Tcl_Sylvatic_^a^	TcI_Dom_^a^	Mix	Not identified
BITER 5	81				55% (45/81)	40% (18/45)	2.2% (1/45)	8.8% (4/45)	48% (22/45)	85.7% (6/7)	50% (3/6)			50% (3/6)
BITER 10										0% (0/2)^b^				
BITER 16		2		1^b^	100% (2/2)	100% (2/2)				50% (1/2)		100% (1/1)		
BITER 17	1		1^b^		100% (1/1)				100% (1/1)					

^a^*Trypanosoma cruzi* DTU I (TcI) genotypes: domestic (TcI_Dom_) and sylvatic (TcI_Sylvatic_)

^b^Animals without *T. cruzi* infection

Triatomines were found to be most abundant in BITER 5 (Aguachica), where we collected 97% of all *R. pallescens* collected in the study (Table 1). Of the 81 *R. pallescens* collected in BITER, 30 and 51 were collected during the rainy and dry seasons, respectively. All bugs captured in the rainy season were in the nymphal state, while 70% (36/51) of insects collected in the dry season were adults. The *T. cruzi* infection rate was higher in triatomine bugs collected in the dry season when compared to those collected in the rainy one (*X*^2^ = 30.22, *P* < 0.0001).

All captured triatomines were evaluated for the presence of *T. cruzi.* Of the* R. pallescens* collected, 56.1% (*n *= 46/82) were positive for *T. cruzi* by the PCR analysis. Two *R. prolixus* were collected, both of which were also infected 100% (2/2). Conversely, *T. dimidiata* and *T. maculata* were not infected (Table 1). TcI genotype was found in all of the* R. pallescens* and *R. prolixus *positive samples corresponding to 57.1% (48/84), with TcI_Sylvatic_ detected in 41.7% (20/48), TcI _Dom_ detected in 2.1% (1/48) and TcI_Dom_/TcI _Sylvatic_ detected in 8.3% (4/48). It was not possible to genotype 47.9% (23/48) of the samples (Table 1).

A total of eight feeding sources were found in 40 specimens of *R. pallescenses* and two of *R. prolixus*. These sources included *D. marsupialis* (opossum) (59.5%, 25/42), *Homo sapiens* (human) (26.2%, 11/42) and other species (14.3%, 6/42): *Metachirus nudicaudatus*,* Mus musculus*,* Philander oposum,*. *Caracara cheriway*, a member of the Cricetidae Family and one individual of the Didelphimorphia Order. Both *R. prolixus* samples evaluated had fed on *Homo sapiens*. All triatomines of both species that had fed on humans were positive for *T. cruzi*, and the only species feeding on birds was also positive for this parasite.

### *Trypanosoma cruzi* infection in synanthropic mammals

Eleven synanthropic mammals were captured, nine *D. marsupialis* and two *Proechimys semispinosus* (Table 1)*.* Seven *D. marsupialis* were captured in BITER 5 (Aguachica), 85% (6/7) of these were positive by molecular detection and hemoculture. The remaining two were captured in BITER 16 (Yopal), one of which was positive by both tools. Only TcI was found in this species. Concerning TcI genotypes, we detected sylvatic TcI in 50% (3/6), and TcI Dom in 16.6% (1/6). However, the 33.3% left (2/6) could not be typed (Table 1). Finally, *P. semispinosus* were not infected.

### *T. cruzi* infection in working and creole dogs

A total of 33 working military dogs (17 male (51%), 16 female (49%)) and 19 creole dogs, present for more than 1 year in the establishments according to the military-staff reports, were evaluated. Of the 33 working dogs, 16 had previously participated in activities involving narcotics and/or explosives detection, while 17 were still being trained and had never been in operational areas. Their age ranged from approximately 8 months to 5 years (median age: 4.5 years). The most common breed was Labrador (*n* = 22), followed by Dutch shepherd (*n* = 7), Belgian shepherd (*n* = 3) and Golden retriever (*n* = 1). The 19 creole dogs (74% male, 16% female; all adult dogs) consisted of stray dogs that were residents of municipalities near the respective BITER. These dogs entered the battalion locations in search of food and were subsequently adopted (Table 2).

**Table 2:** Frequency of seropositivity with *T. cruzi* in military working dogs and creole dogs in four BITERs in three municipalities of Colombia between 2019 and 2020

Municipalities (BITER)	Working dogs, sample size (seropositive %)	Creole dogs, sample size (seropositive %)
Aguachica (BITER 5)	2 (0%)	7 (43%)
El Yopal (BITER 16)	2 (0%)	7 (14%)
Carepa (BITER 17)	29 (3.44%)	0
La Loma (BITER 10)	0	5 (0%)
Total	3.03%	21.05%

Of the 52 dogs sampled, five (9.6%, 95% confidence interval 95% 3.20–21.03) were seropositive based on two independent tests. Four of the dogs testing positive were creole, with the following distribution: three from BITER 5 (Aguachica) and one from BITER 16 (El Yopal). The one seropositive working dog was detected in BITER 17 (Carepa) and has served in explosive detection activities inside the operational area. No infection was detected in the dogs from BITER 10 (La Loma) (Table 2). Molecular diagnosis of blood samples stored in guanidine chloride buffer (BCG), which amplified the satellite DNA of *T. cruzi,*, did not show the presence of parasites in the 52 dogs evaluated.

### Geospatial analysis

**Fig. 2:**
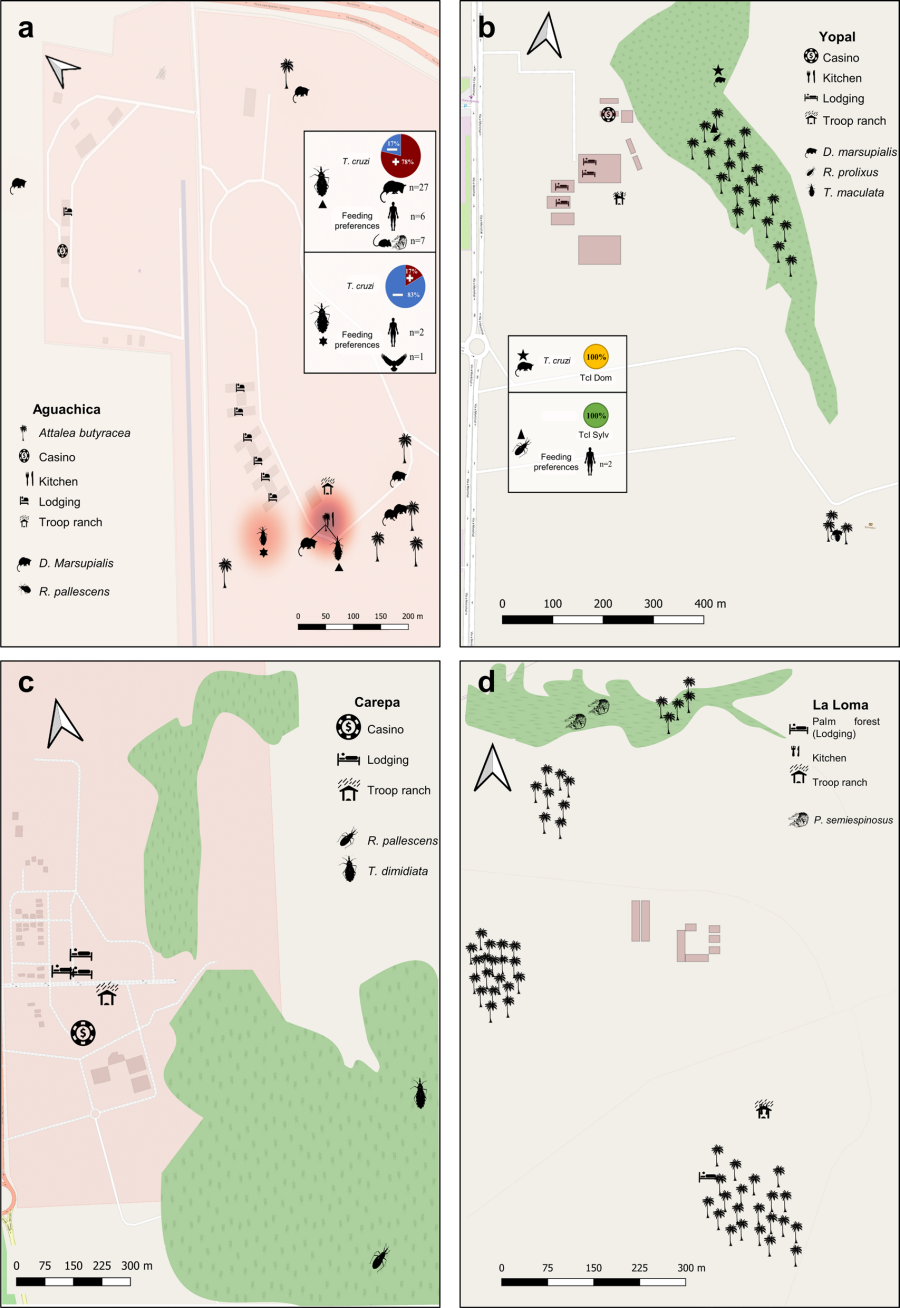
Geospatial distribution of triatomines and host (infected and not infected) in four BITER battalions in Colombia. **a** BITER 5 (Aguachica), **b** BITER 16 (Yopal), **c** BITER 17 (Carepa), **d** BITER 10 (La Loma). Maps were prepared using shapefiles from OpenStreetMap Standard

Analysis of the generated maps showed areas inside the battalions with a greater risk of transmission due to the abundance of infected triatomines and synanthropic mammals (Fig. 2). The presence of infected *R. pallescens* that fed on human blood and of infected *D. marsupialis* near the lodgings and kitchens suggests a potential risk of vectorial and oral transmission of *T. cruzi* in BITER 5 (Aguachica) and BITER 16 (Yopal). A lower potential risk was observed in BITER 10 (La Loma), where infected triatomine bugs were not found near the location, and BITER 5, where no *T. cruzi* infections were found in synanthropic mammals (Fig. 2).

## Discussion

The success of the Southern Cone Initiative (INCOSUR), which achieved the elimination of *T. infestans* in many areas and the elimination of *R. prolixus* in some Central America countries and some Colombian municipalities, is important in terms of the significant advances in CD control [52, 53]. However, the enzootic or sylvatic cycle in triatomines across all continents, together with the wide distribution of household-invading triatomines, is now responsible for most CD transmission in countries like Brazil, Argentina, Colombia and Nicaragua [14, 17, 18, 31, 32]. A scenario where the restriction of a given multi-host parasite based on the control of one single vector species will always be insufficient because parasite transmission rarely relies on a single system [27, 54]. Therefore, the sustainability of successful control strategies for CD requires more accurate knowledge of environmental factors that underlie the enzootic transmission cycle of this parasite [55]. In Colombia, aspects determining the current epidemiology of CD are still unknown, and the potential risk of enzootic transmission inside overcrowded settlements and installations, such as work camps or military barracks, located in endemic areas has not been explored. Here we described the potential risk for spillover infections in the military population by a well-established *T. cruzi* enzootic cycle due to: (i) a high rate of *T. cruzi* infection of triatomine bugs collected; (ii) increased contact between *T. cruzi-*infected triatomines and humans according to blood source analysis; (iii) selection and dominance of mammalian species with high competence for being a reservoir; and (iv) close proximity of infected triatomines and infected *D. marsupialis* to accommodations and kitchens (Table 1; Fig. 2).

Determining the spatial distribution of the elements that form this epidemiological chain in the enzootic cycle and suitable contact with humans are of pivotal importance for determining trends and risk evaluation. The present study was developed in four BITERs located in four municipalities with historical reports of infected triatomine bugs [36, 37]. However, differences in potential risk intensities of *T. cruzi* transmission were found at these locations. BITER 10 (La Loma) and 17 (Carepa) could be considered to have a lower potential risk of *T. cruzi* transmission, while BITER 5 (Aguachica) and 16 (Yopal) display comparable patterns regarding the presence of naturally *T. cruzi*-infected *R. pallescens* and *R. prolixus*, respectively. These patterns include the positive selection of generalist mammal species with high transmissibility competence, such as *D. marsupialis*. These eco-epidemiological characteristics could increase the opportunities for contact between humans, domestic animals and the sylvatic cycle. These findings in BITERs with a higher potential risk are in accordance with the epidemiological situation reported by the SIVIGILA for these areas (Fig. 1) and agree with previous outbreaks of CD caused by oral transmission due to spillover of the enzootic cycle described in recent years in the municipalities of Aguachica, Yopal and Paz de Ariporo (Casanare) [18, 56, 57].

BITER 5, located in Aguachica municipality in the Caribbean region of Colombia, presents an epidemiological profile that enhances the chances of transmission to humans, thereby representing a potential risk for around 30,000 active members of the National Army who participate in training and re-training activities each year. The results of this study show a close proximity between the enzootic transmission cycle and the quartered population. Molecular analysis supports the notion that *R. pallescens* actively participates in the enzootic transmission, showing the high-frequency infection with the TcI_Sylvatic_ (46.8%) genotype and the high frequency of feeding on *D. marsupialis* (66%) and other sylvatic mammals. However, the presence of human blood in 18.6% of the analyzed insects and 11.45% infection with TcI_Dom_, reveal an active interplay between sylvatic and domestic transmission, as previously reported in other regions of Colombia [18]. The same ecological situation was found in BITER 16 where we found *R. prolixus* in *A. butyracea* palms infected with TcI_Sylvatic_ that fed on human blood, which can lead to a potential risk of *T. cruzi* transmission to more than 30,000 active members present in the Orinoco region. These findings are in agreement with those of previous studies and support the premise that in Colombia there is a high risk of *R. pallescens* intrusion supported by the high presence blood-feeding on humans and *T. cruzi* infection [18].

The spatial convergence found in the BITER 5 location facilitates triatomines reaching human installations by being attracted by artificial light in the search of food [17, 58]. A similar eco-epidemiological situation has been described in some regions of Colombia and Panama where this species has been found to be a *T. cruzi* vector [16, 38, 59, 60]. The higher number of triatomines found in the dry season added to the higher infection rates in this season, increasing the risk of an oral outbreak in this period [17, 18, 56]. These ecological characteristics reveal the ability of *R. pallescens* to participate in complex transmission dynamics that may exhibit local peculiarities and lead to the emergence or re-emergence of outbreaks.

On the other hand, the dominance of *D. marsupialis* with high transmissibility, as shown by hemoculture, supports the high potential risk inside BITER 5 and 16. The reduced diversity and the selection of suitable reservoir hosts of *T. cruzi* have been reported as common traits and possible factors related to human outbreaks by the oral route [27, 54]. In these military installations, the sympatry of the common opossum *R. pallescens* and* R. prolixus* and humans near the kitchen enhanced the risk of oral transmission through the consumption of food or juices contaminated by triatomine feces or food/beverages contaminated with animal secretions that contain metacyclic trypomastigotes [32, 61, 62]. The outbreaks due to accidental food contamination in these BITERs would be challenging to manage due to the large number of people affected. Future studies should include high-resolution molecular tracking to unravel the transmission dynamics of *T. cruzi* infection within these settlements.

BITER 17 and 10, located in Carepa (Antioquia) and La Loma (Cesar), respectively, were found to have the lowest potential risk of CD transmission. In the Uraba region of BITER 17, we collected only two triatomine bugs: one infected *R. pallescens* and one non-infected *T. dimidiata*. Contrary to BITER 5 and 16, triatomines captured in this BITER were found by military personnel in forest areas far away from the installations inhabited by military personnel (Fig. 2). These results show the existence of enzootic transmission with *R. pallescens*. Regarding *T. dimidiata,* metapopulations of this species distributed in the Urabá region are considered to be of low epidemiologic relevance [63, 64]. Finally, we believe that we did not capture mammals inside this military installation due to the ready availability of banana crops around the BITER [65]. All these factors support a low potential risk of *T. cruzi* in this area.

In addition, no triatomine bug was collected in BITER 10, and even the two potential reservoirs (*P. semiespinosus*) collected there were not infected [55]. BITER 10 is in La Loma, a rural area of the municipality of La Jagua de Ibirico (Cesar), a zone with a high abundance of *A. butyracea* palms and reports of vectors, such as *R. pallescens*,* Erathyrus cuspidatus* and *T. dimidiata* with their ecotopes in this palm species [36, 37]. Our results show the absence of triatomine bugs in palms near the bedrooms and kitchen of BITER 10, but the enzootic cycle could be happening in palms forest not included in the present study. However, it is necessary to mention that BITER 10 is in an area of high mining exploitation and accelerated desertification processes where environmental characteristics, such as vapor pressure, can affect the abundance of stenohydric species like *R. pallescens* [65, 66]. We suggest that both hypotheses should be evaluated in the future studies.

The serological evaluation of the working dogs showed that one of the working military dogs was infected with *T. cruzi*. In the Colombian National Army the function of the guard dog is fundamental for troop protection in mission activities, where both soldiers and guard dogs remain for long periods within sylvatic areas. These activities have been related to the infection of working army dogs with canine cutaneous leishmaniasis in Colombia [60]. To our knowledge, few studies have explored the CD infection levels in dogs of Latin America. Here, we reported a *T. cruzi* infection in military working dogs of Colombia. However, the epidemiological relevance and the veterinarian importance of infected dogs should be determined in the future. Finally, the presence of infected stray dogs inside the studied BITER is a finding that draws our attention due to the known role of domestic dogs as synanthropic reservoirs that could generate new transmission foci inside BITERs [67–70].

There are some limitations to the present study. First, the results are not supported by a seroprevalence study in the military population present in the four BITERs evaluated. This aspect could be considered in the future. Second, Colombia’s National Army has 14 BITERs, with many located in municipalities with ecological conditions for *T. cruzi* transmission and other pathogens transmitted by insects, such as leishmaniasis, malaria and dengue. We did not include these BITERs in the present study nor did we assess the risk of infection of other diseases transmitted by insects. Future studies should expand the number of BITERS included in the analysis.

This study provides new insightful information on *T. cruzi* transmission dynamics and the potential risk of oral transmission in an area with non-domiciled vectors in some locations in Colombia, including the presence of triatomines adapted to *A. butyracea* forests and coexisting with synanthropic mammal reservoirs as *D. marsupialis*. These scenarios require an alternative eco-health (One Health) approach for reducing human–triatomine bugs and human–*D. marsupialis* contact through: (i) the managing the environment by eliminating palms near lodgings and kitchens; (ii) proper care of area inside battalion or cantonment centers; and (iii) proper waste management. Finally, it is necessary to achieve active community participation with the military population, an entomological surveillance program led by the staff of each battalion and health educational during the training and re-training activities as a sustainability strategy including regular testing for CD.

## Conclusions

In conclusion, we present a study in four training and re-training battalions (BITERs) where around 100,000 active Colombian military troops receive training over the years. The evaluation of *T. cruzi* natural infection in mammals, added to the identification of blood-meal sources in triatomine bugs, genotyping of *T. cruzi* and results of the spatial analysis, provide a better understanding of the parasite’s spillover process and potential risk of infection for the military population inside these military camps. The same scenario may be relevant to other military establishments of Colombia and other endemic countries. Therefore, these conditions should be evaluated in all military and working camps located in endemic areas. Future serological and molecular studies among military population in BITERs with the highest potential risk should be carried out to establish in deeper detail the extent of human infection and the circulation of *T. cruzi* in light of the findings of the present study.

## Supplementary Information

**Additional file 1: Appendix S1.** Primers and thermal profiles condition for the genotyping of *T. cruzi.*

**Additional file 2: Figure S1.** Algorithm of *T. cruzi* genotyping in positive samples.

## Data Availability

All data sets used or analysed in the current study are included in the supplementary information files of this published article. All genetic sequences derived of current study are deposited and available in GenBank (https://www.ncbi.nlm.nih.gov/genbank/) under accession numbers OK040172 - OK040196 and OK058390 - OK058403.

## Abbreviations

BCG: Guanidine hydrochloride buffer
BCEI: Grupo Biología y Control de Enfermedades Infecciosas
BITER: Battalion of Instruction, Training en Re-training
BLASTn: Basic local alignment search tool for nucleotide databases
CD: Chagas disease
CIMBIUR: Centro de Investigaciones en Microbiología y Biotecnología de la Universidad del Rosario
ExoSAP-IT: Exonuclease I and shrimp alkaline phosphatase
DTU: Discrete typing unit
ELISA: Enzyme-linked immunosorbent assay
GINETEJ: Grupo de Investigación en Enfermedades Tropicales del Ejército
IFAT: Indirect immunofluorescence antibody test
INCOSUR: Southern Cone Initiative
GPS: Global Positioning System
IgG: Immunoglobulin G
MG-DANE: National Administrative Department of Statistics
NCBI: National Center for Biotechnology Information
rRNA: Ribosomal ribonucleic acid
SL-IR: Spliced leader intergenic region
SIVIGILA: National Public Health Surveillance System
TcI: *Trypanosoma cruzi* DTU I
WGS: World Geodetic System
