# Supplementary material for: The potential risk of enzootic Trypanosoma cruzi transmission inside four training and re-training military battalions (BITER) in Colombia
Source: Parasit Vectors. 2021 Oct 9;14:519. doi: 10.1186/s13071-021-05018-4 (PMC8501693; doi:10.1186/s13071-021-05018-4)
Supplement: Supplementary file 1 — Additional file 1: Appendix S1. Primers and thermal profiles condition for the genotyping of T. cruzi. [file 13071_2021_5018_MOESM1_ESM.docx]

**Genotyping of *T. cruzi***

**Primers and thermal profiles condition**

- **(SL-IR)**

| **Primer** | **Sequence** |
| --- | --- |
| **TCC** | 5´- CCCCCCTCCCAGGCCACACTG-´3 |
| **TC1M** | 5´- GTGTCCGCCACCTCCTTCGGGCC-´3 |
| **TC2** | 5´- CCTGCAGGCACACGTGTGTGTG-´3 |

| **Thermal profile SL-IR** | | |
| --- | --- | --- |
| **Temperature** | | **Time** |
| **95°C** | | 5 min |
| **5 CYCLES** | | |
| **94°C**  **67°C**  **72°C** | | 1 min  1 min  1 min |
| **5 CYCLES** | | |
| **94°C**  **65°C**  **72°C** | | 1 min  1 min  1 min |
| **5 CYCLES** | | |
| **94°C**  **63°C**  **72°C** | 1 min  1 min  1 min | |
| **30 CYCLES** | | |
| **94°C**  **61°C**  **72°C** | | 1 min  1 min  1 min |
| **FINAL EXTENSION** | | |
| **72°C** | 10 min | |

- **24Sα**

| **Primer** | **Sequence** |
| --- | --- |
| **D71** | 5´-CCCCCCTCCCAGGCCACACTG -´3 |
| **D72** | 5´- GTGTCCGCCACCTCCTTCGGGCC -´3 |

| **THERMAL PROFILE 24Sα** | | |
| --- | --- | --- |
| **Temperature** | | **Time** |
| **94°C** | | 5 minutes |
| **30 cycles** | | |
| **94 °C** | | 1 minute |
| **60°C** | | 30 seconds |
| **72°C** | | 1 minute |
| **1 cycle** | | |
| **72°C** | 5 minutes | |
| **4°C** | Infinite | |
