# Supplementary figures and images for: The potential risk of enzootic Trypanosoma cruzi transmission inside four training and re-training military battalions (BITER) in Colombia
Source: Parasit Vectors. 2021 Oct 9;14:519. doi: 10.1186/s13071-021-05018-4 (PMC8501693; doi:10.1186/s13071-021-05018-4)

## Genotyping of *T. cruzi* in positive samples

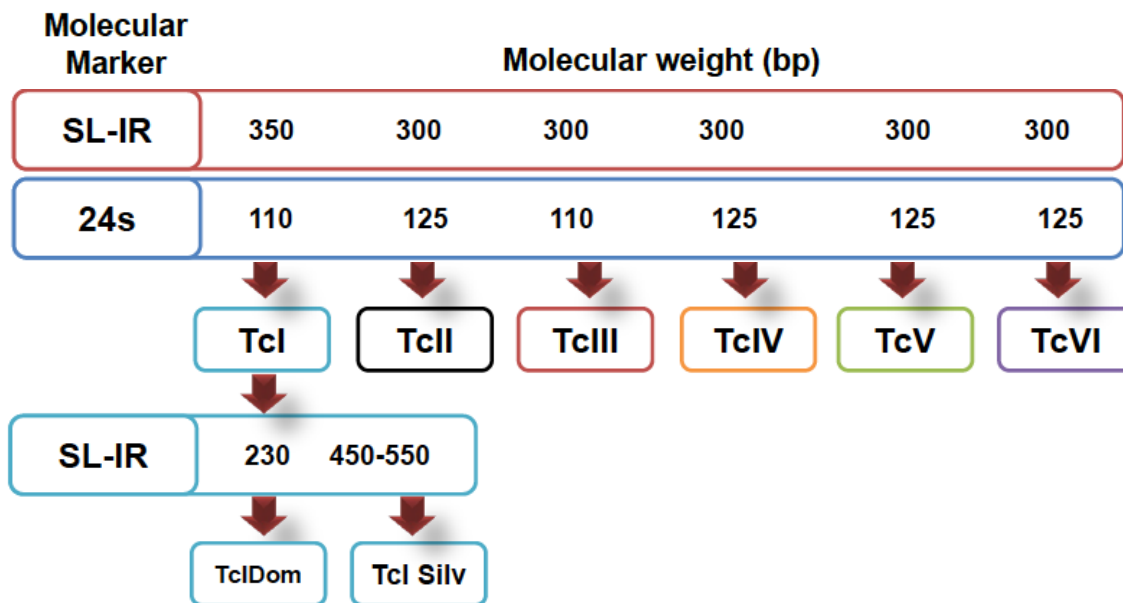

Adapted of Hernandez et al 2016

Supplement: Supplementary file 2 — Additional file 2: Figure S1. Algorithm of T. cruzi genotyping in positive samples. [file 13071_2021_5018_MOESM2_ESM.pdf]
